# Reduction in broad-spectrum antimicrobial prescriptions by primary care pediatricians following a multifaceted antimicrobial stewardship program

**DOI:** 10.3389/fped.2022.1070325

**Published:** 2023-01-06

**Authors:** F Pagano, C Amato, G De Marco, M Micillo, G Cecere, M Poeta, A Guarino, A Lo Vecchio

**Affiliations:** ^1^ Department of Translational Medical Sciences, Section of Pediatrics, University of Naples Federico II, Naples, Italy; ^2^ U.O. Materno Infantile, ASL Napoli 1 Centro, Distretto Sanitario 28, Naples, Italy

**Keywords:** antibiotic, antimicrobial resistance, antimicrobial stewardship, primary care, pediatrics, prescription

## Abstract

**Background:**

Since 2016, following the Italian “National Plan to Contrast Antimicrobial Resistance”, Campania Region has implemented an antimicrobial stewardship program, including the obligation to associate an appropriate International Classification of Diseases-9 code to each antibiotic prescription, the publication of schemes for empirical antibiotic therapy and educational interventions.

**Methods:**

To evaluate the impact of these interventions on the prescribing habits of family pediatricians, we conducted a retrospective cohort study (January 2016–December 2020), including all patients registered in an associate practice of Primary Care Pediatricians. We collected data on antibiotic prescriptions through a specific study management software; our primary outcomes were the annual prescription rates, calculated for both the number of patients in follow-up and the number of medical consultations, and the annual prescription rates for selected antibiotic classes and molecules. To investigate the hypothesis that chronic conditions would be associated with an increased rate of prescription, we also tested the association between underlying conditions and the number of antibiotics received.

**Results:**

During the study period, 2,599 children received 11,364 antibiotic prescriptions (mean 4.37, SD 4.28). From 2016 to 2020 we observed a substantial reduction in both the annual prescription rate per 100 patients (9.33 to 3.39; *R*^2 ^= 0.927, *p* = 0.009), and the annual prescription rate per 100 medical consultations (25.49 to 15.98; *R*^2 ^= 0.996, *p* < 0.01). The prescription rates of Amoxicillin-Clavulanate (50.25 to 14.21; *R*^2 ^= 0.983, *p* = 0.001) and third generation Cephalosporins (28.43 to 5.43; *R*^2 ^= 0.995, *p* < 0.01) significantly decreased; we didn't find significant modifications in the prescription rates of Amoxicillin and Quinolones; finally, we observed a trend toward reduction in the prescription of Macrolides. No statistical association was found between antibiotics prescribing frequency and history of chronic diseases.

**Discussion:**

Following the implementation of the regional interventions on antimicrobial stewardship, we observed a substantial reduction in the overall antibiotic prescription per patients and per medical consultations, with a statistically significant reduction in the use of broad-spectrum molecules. Considering the results of our analysis, new guidance and training interventions addressed to specialists in the primary care sector should be implemented to further limit antibiotic resistance.

## Introduction

Antimicrobial Resistance (AMR) is still a matter of concern for health organizations worldwide, because of its burden both on public health and patients' management. Direct consequences of infections by multidrug resistant (MDR) pathogens include prolonged illness and hospital stay, increased complications and mortality, and loss of protection for a growing number of fragile patients. On a large scale this phenomenon is causing a relevant raise in healthcare expenditure for Western countries, directly involved in the selection of resistant strains, while developing countries are increasingly exposed to the risk of circulation of MDR bacterial species without having the possibility of acquiring new generation molecules (1).

Despite the fact that an overall improvement has been reported in the last few years, data on antibiotic consumption and AMR in Italy are alarming. According to the European Surveillance of Antimicrobial Consumption Network (ESAC-Net), Italian quality indicators for antimicrobial prescription in the community are worse than the European average, including the consumption of antibiotics for systemic use, combination of Penicillin and Beta-lactamase inhibitor, third and fourth generation Cephalosporins and the ratio between the consumption of broad-spectrum and narrow-spectrum Penicillins, Cephalosporins and Macrolides (2). As a consequence, the Italian percentage of MDR antimicrobial isolates registered by the European Antimicrobial Resistance Surveillance System (EARSS) is above the European average, only surpassed by Croatia, Greece, Lithuania and Romania, which reflects a striking gap in AMR indexes between Northern countries and the Southern and Eastern Europe (3). Indeed, there is a well-documented correlation between levels of outpatient antibiotic prescriptions - in particular of broad-spectrum molecules - and AMR indexes, accounting for different selective pressure in various geographic areas. Community antibiotic consumption is a crucial driver of resistance emergence both in inpatient and outpatient settings (4). Magee et al. reported a correlation between antibiotic resistance in coliform organisms in urine samples and the use of antibiotics by a general practice in Wales, UK (5).

Goossens et. al reported a high AMR rate in countries with high consumption of antibiotics (6). This association between antimicrobial drug consumption patterns and AMR has been well studied for specific pathogens. Resistant strains of Streptococcus Pneumoniae, whose infections are more frequent in children than in adults, have been associated with an overprescription of Penicillins and Macrolides in pediatric age (7–9).

AMR rates are higher in Southern and Eastern Europe, where generally more antibiotics are consumed, than in Northern European countries (2, 3, 6, 10). There is also a high variability among Italian regional data, reporting a greater antibiotic consumption in the Southern and Central regions compared to the Northern. However, a progressive trend towards a more judicious use of broad-spectrum antimicrobials has been observed in the areas of largest use. Specifically, Campania region and Sardinia region are recording the highest contraction in antibiotic consumption, in recent years (10, 11). The largest antibiotic use habit is reported in the primary care rather than in hospital settings and is associated with the pediatric management of the upper respiratory tract infections (URTI). This pattern is similar to the one reported in many other geographical and professional settings although these prescriptions are often inappropriate and poorly effective in improving patient's clinical outcomes (12).

For all these reasons, in the past twenty years, antimicrobial stewardship programs have been implemented both at European, national, and regional level, in order to limit the number of inappropriate prescriptions. Although it is established that pediatric Antimicrobial Stewardship programs have a significant impact on the reduction of antibiotic use, healthcare costs, and antimicrobial resistance in both inpatient and outpatient settings, the most effective intervention in cutting antibiotic prescriptions is not yet clearly identified (13, 14).

We hereby report the impact of a multifaceted intervention implemented at regional level on the prescribing habits of family pediatricians, which are a primary target of local antimicrobial stewardship programs.

## Methods

### Study design and setting

We conducted a retrospective open cohort study (January 2016–December 2020), including all patients aged 0–16 years registered in an associate practice of Primary Care Pediatricians (PCPs) located in a single Sanitary District in the province of Naples, the largest city in Southern Italy. The district accounts for about ninety thousand inhabitants and covers a surface of 17.45 Km^2^, comprising some urban areas among those with the highest population density and the lowest-medium socioeconomic status in the metropolitan area.

The associate practice taking part in the study is composed of three PCPs responsible for about three thousand patients living in the area. In the Italian healthcare system PCPs provide a first-line access for health issues to all children at no cost and drive patients to further access to care if needed. PCPs are in charge of the promotion of health, primary and secondary prevention, first approach to diagnosis and treatment of acute conditions and follow-up of patients with chronic diseases.

### Regional antimicrobial stewardship program

Starting from 2016, the Campania Region endorsed the principles defined by the “National Plan to Contrast Antimicrobial Resistance” (PNCAR) (15) and started implementing a bundle of interventions to promote a judicious use of antimicrobials in adults and children (Table 1).

**Table 1:** Summary of regional antimicrobial stewardship interventions.

Year	Intervention	Content
2016	Mandatory ICD-9 code for each antibiotic prescription	Obligation for Primary Care Physicians to associate an appropriate diagnosis, encoded according to the ICD-9 classification, to each antibiotic prescription
2017	Technical committee for surveillance on HAI risk and AMR	Support function to the actualization of PNCAR in the Campania Region
2018	Guidelines for the implementation of AMS program and regional protocols on antibiotic therapy	Schemes of empiric antibiotic therapy developed by a multidisciplinar work group on AMS and monitoring of prescriptive appropriateness, including: - microbiologists - pharmacists - epidemiologists - infectious disease specialists - pediatric infectious disease specialists - primary care physicians - primary care pediatricians
2019	E-learning course on Antibiotic Therapy and AMS addressed to Healthcare Professionals (biologists, chemists, pharmacists, nurses, medical doctors, obstetricians, veterinarians)	Two modules out of thirteen were specifically addressed to Pediatric care including illustrative clinical scenarios: Module I: - Antibiotic consumption in the pediatric population - Determinants of antibiotic prescription in the pediatric age - Principles of Antibiotic Therapy for common pediatric infections (URTI, otitis, pharyngitis, pneumonia) - Self-help tools for rapid diagnosis - Strategies for judicious use of antibiotics in the pediatric age Module II: - Principles of pharmacokinetics of commonly used molecules in the pediatric age - Strategies for judicious use of antibiotics in the pediatric age - Severe bacterial infections in inpatient settings - Other bacterial infections in outpatient settings in the pediatric age

Firstly, all primary care physicians were asked to associate an appropriate International Classification of Diseases-9 (ICD-9) code to each antibiotic prescription (16). In 2017, a regional technical committee for surveillance on hospital-acquired infections (HAI) risk and AMR was set up to support the implementation of PNCAR in Campania. The committee implemented schemes for empirical antibiotic therapy for adults and children (17) and developed an online educational package addressed to primary care physicians (general practitioners, community pediatricians). In line with the European strategy for surveillance of MDR pathogens, the committee reviewed and empowered a previously active epidemiological surveillance system for invasive infections caused by eight MDR strains (E. coli, K. pneumoniae, P. aeruginosa, A. baumannii, S. pneumoniae, S. aureus, E. faecalis, E. faecium) isolated in blood or cerebrospinal fluids. This system is based on the collection of samples from patients in ordinary hospitalization, outpatient access or daytime regimen that are analyzed in twenty-five laboratories participating in the network. This system allows estimating the local AMR rates to inform practitioners and support the empirical antibiotic protocols.

All pediatricians who took part in the present study participated in each phase of the program: acknowledged the obligation to associate diagnostic codes to antibiotic prescription, received the regional indications for empiric antibiotic treatment for the most common pediatric infections, and finally attended the online e-learning classes.

### Data collection

The software Kappamed (version 12.4.30), routinary used by the study pediatricians to record and manage patients' personal details, medical history, diagnostic work-up and treatment, was used to retrospectively obtain data about antibiotic prescriptions. At the time of the first consultation with the pediatrician, a parent or legal guardian was asked to provide an informed consent to the processing of the patient's personal data, according to local procedures and to the Declaration of Helsinki. Although this analysis did not require a formal approval by the Ethical Committee, all data regarding antibiotic prescriptions by each pediatrician during the study period were extracted from the software and linked to an anonymous alphanumeric code for each patient. All the prescriptions were then merged into the same database and rearranged by the alphanumeric code in order to allocate multiple prescriptions to each patient during the follow-up period.

The collected data included: patient's alphanumeric code, date of birth, gender, number of siblings, Body Mass Index (BMI), date of prescription, age at the time of the prescription, antibiotic class and molecule, single diagnosis and previous health problems encoded according to ICD-9.

### Statistical analysis

Data are presented as mean ± standard deviation (SD), median and interquartile range (IQR) and proportions are expressed as percentage (%). The statistical analysis was performed using IBM SPSS Statistics, released 28.0.1.1. Categorical variables were compared using the Chi-2 test and Fisher's exact test. Continuous variables were compared using the unpaired Student's t-test or Mann Whitney, as appropriate. The variation of the rates during the study period was analyzed with a linear regression test. A *p*-value of less than 0.05 was considered significant.

Our primary outcome was the annual prescription rate calculated for both the number of patients in follow-up and the number of medical consultations per year. We used the number of medical consultations as a denominator in order to minimize the potential impact of COVID-19 pandemic, which could have resulted in a decrease in prescription rates, due to a limited access to medical care during the pandemic.

As a secondary outcome we calculated the annual prescription rates and percentage of specific classes and molecules, focusing on the broad-spectrum antibiotics most widely prescribed.

In addition, we compared the prescription of antibiotics in the four years before the beginning of the COVID-19 pandemic to the consumption reported in 2020, in order to estimate a possible change in prescription habits during pandemic. Specifically, we analyzed the prescription rates of Amoxicillin, Amoxicillin-Clavulanate, Clarithromycin, Azithromycin, third generation Cephalosporins and Quinolones.

We calculated and analyzed the trend over time of the annual Amoxicillin/Amoxicillin-Clavulanate index and *Access/Watch* index. The latter is based on the *AWaRe* classification by the World Health Organization (WHO), that categorizes antibiotics in three groups according to the risk of AMR selection:

- *Access*, which includes antibiotics that have activity against a wide range of commonly encountered susceptible pathogens while showing lower resistance potential than antibiotics in the other groups. This group includes antibiotics that are recommended as essential first or second choice empiric treatment options.
- *Watch*, which includes antibiotic classes that are at relatively high risk of selection of bacterial resistance. These medicines should be prioritized as key targets of stewardship programs and monitoring.
- *Reserve*, which includes antibiotics that should be reserved for treatment of confirmed or suspected infections due to multidrug-resistant organisms.

Outpatients prescriptions recorded during the study period did not include antibiotics classified as *Reserve*, that are usually prescribed in inpatient setting, but only antibiotics classified as *Access* (Amoxicillin, Amoxicillin-Clavulanate, Ampicillin, Ampicillin-Sulbactam, Benzylpenicillin, Clindamycin and Trimethoprim-Sulfamethoxazole) and *Watch* (Piperacillin-Tazobactam, second and third generation Cephalosporins, Lincomycin, Teicoplanin, Azithromycin, Clarithromycin, Tobramycin, Quinolones, Tetracycline and Fosfomycin) according to the AWaRe classification (18).

We investigated a seasonal pattern in the monthly prescription rate/100 patients by performing a multiple regression analysis, with month and year as dummy variables. Months from April to September were classified as “Summer”, while months from October to March were classified as “Winter”. Each year included in the analysis started in July and ended in June of the following calendar year; years were then categorized into sequential time points (e.g., 1 = 2016, 2 = 2017).

Finally, we tested the association between the annual number of prescriptions received by each child and the presence of underlying chronic conditions, including risk factors for recurrent urinary tract infections (UTI) (i.e., vescico-ureteral reflux, hydronephrosis, congenital defects of the urinary tract), overweight and obesity, asthma, prematurity, chronic tonsillar diseases, lymph-adenopathy, previous episodes of otitis media, bronchitis, pneumonia, autism and developmental delay. We investigated the association between these conditions and the possibility to receive a number of antibiotic prescriptions higher than median value.

Children who received a number of antibiotic prescriptions 3 or more IQR higher than the median were identified as *outliers.* The presence of the health conditions above mentioned was investigated in these patients as well.

## Results

### Study population and overall prescription rates

Overall, 2,599 children (F 48.74%, median age 5.43 years, range 0–16) were included in the study. Only 458 (17.6%) had one or more siblings. Regarding underlying conditions and past medical history, 996 (38.4%) children were overweight or obese, 1,025 (39.4%) had previous episodes of respiratory tract infections, including otitis media, bronchitis and pneumonia and 59 (2.3%) had risk factors for recurrent UTI (Table 2).

**Table 2:** Descriptive analysis of population.

Total number of children	2599
Age, y, median (IQR)	5.43 (6.21)
Female gender, *n* (%)	1,267 (48.74)
Number of siblings, *n* (%)	
0	2,141 (82.40)
1	313 (12.00)
2	140 (5.40)
3	3 (0.10)
4	2 (0.10)
BMI, *n* (%)	
< 85° pc	1,370 (52.70)
85°–95° pc	628 (24.20)
>95° pc	368 (14.20)
Unknown	233 (9.00)
History of previous infections, *n* (%)	
Otitis media	509 (19.60)
Bronchitis	341 (13.10)
Pneumonia	175 (6.70)
Risk factors for recurrent UTIs	59 (2.30)
Number of children receiving antibiotic, *n* (%)	
1–5 prescriptions	1,940 (74.64)
6–10 prescriptions	437 (16.81)
11–15 prescriptions	154 (5.93)
>15 prescriptions	68 (2.62)

**Figure 1:**
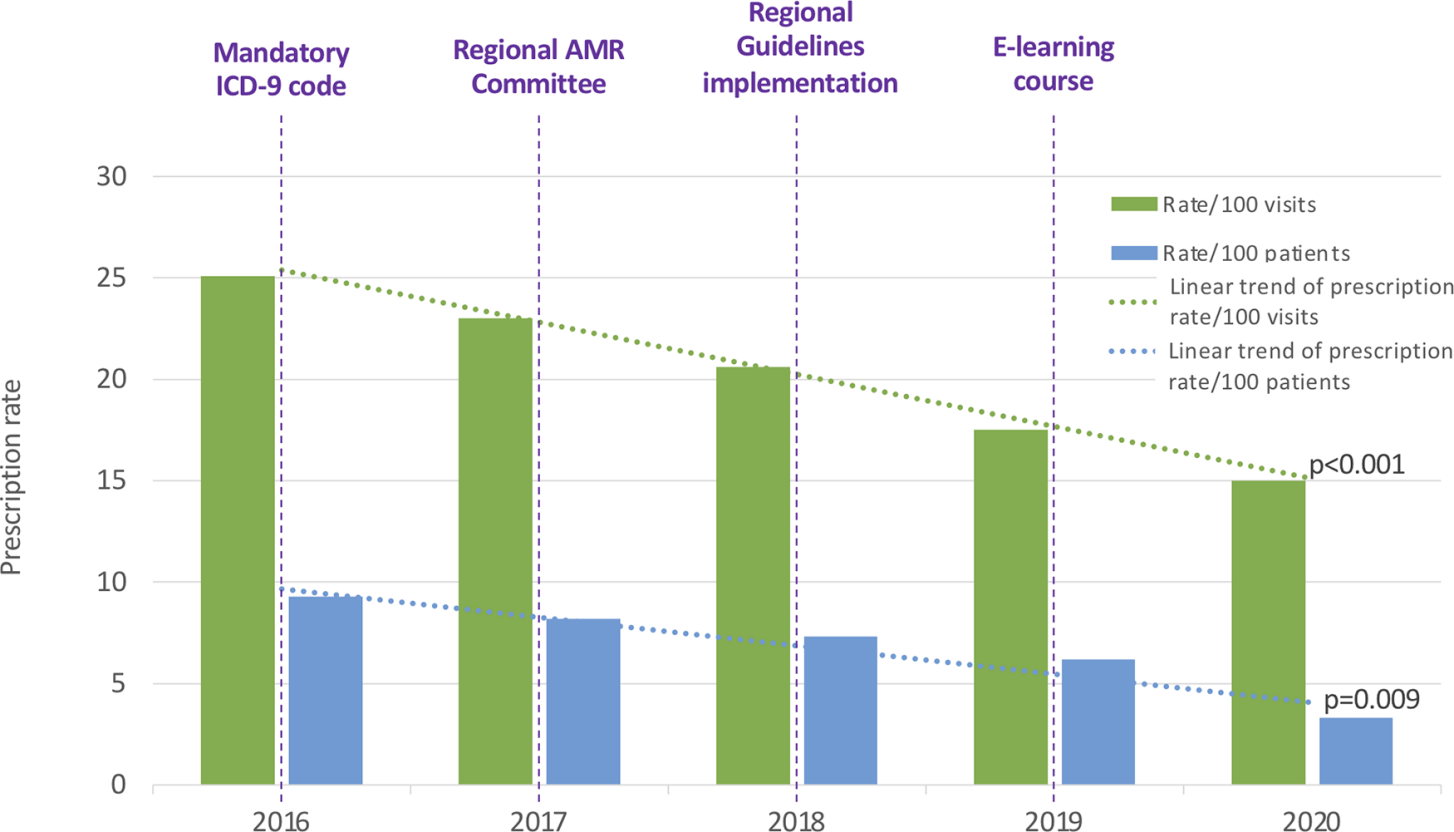
Annual antibiotic prescription rates per 100 patients and 100 visits. ICD-9: International Classification of Diseases-9. AMR, Antimicrobial Resistance.

We observed a significant reduction of the overall antibiotic prescriptions in both the annual prescription rate per 100 patients (9.33 to 3.39; *R*^2 ^= 0.927, *p* = 0.009), and the annual prescription rate per 100 medical consultations (25.49 to 15.98; *R*^2 ^= 0.996, *p* < 0.001) (Figure 1).

### Trend of broad-spectrum antibiotics prescription rates

Overall, 11,364 antibiotic prescriptions (mean 4.37, SD 4.28) were recorded in the 2,599 children. The distribution and characteristics of annual antibiotic prescriptions is reported in Table 3.

**Table 3:** Antibiotic prescriptions distribution and characteristics.

Year	2016	2017	2018	2019	2020	Tot
Antibiotic prescriptions, *n*	3107	2699	2401	2064	1093	11,364
Median (IQR)	2 (2)	2 (2)	2 (2)	2 (2)	2 (2)	3 (4)
Molecule/Class, *n* (%)						
Amoxicillin	309 (9.95)	256 (9.48)	289 (11.07)	688 (33.33)	360 (32.94)	1,902 (16.74)
Amoxicillin-Clavulanate	1,395 (44.90)	1,237 (45.83)	962 (40.07)	690 (33.43)	382 (34.95)	4,666 (41.07)
Cephalosporins, 3rd gen	789 (25.39)	614 (22.75)	474 (19.74)	274 (13.28)	146 (13.36)	2,297 (20.22)
Macrolides	484 (15.58)	516 (19.12)	529 (19.53)	340 (12.35)	166 (6.17)	2,035 (17.91)
Azithromycin	118 (3.80)	106 (3.93)	95 (3.96)	31 (1.5)	38 (3.48)	388 (3.41)
Clarithromycin	366 (11.78)	410 (15.19)	434 (18.07)	309 (14.97)	128 (11.71)	1,647 (14.49)
Quinolones	8 (0.26)	11 (0.41)	22 (0.92)	7 (0.34)	2 (0.18)	50 (0.44)

**Figure 2:**
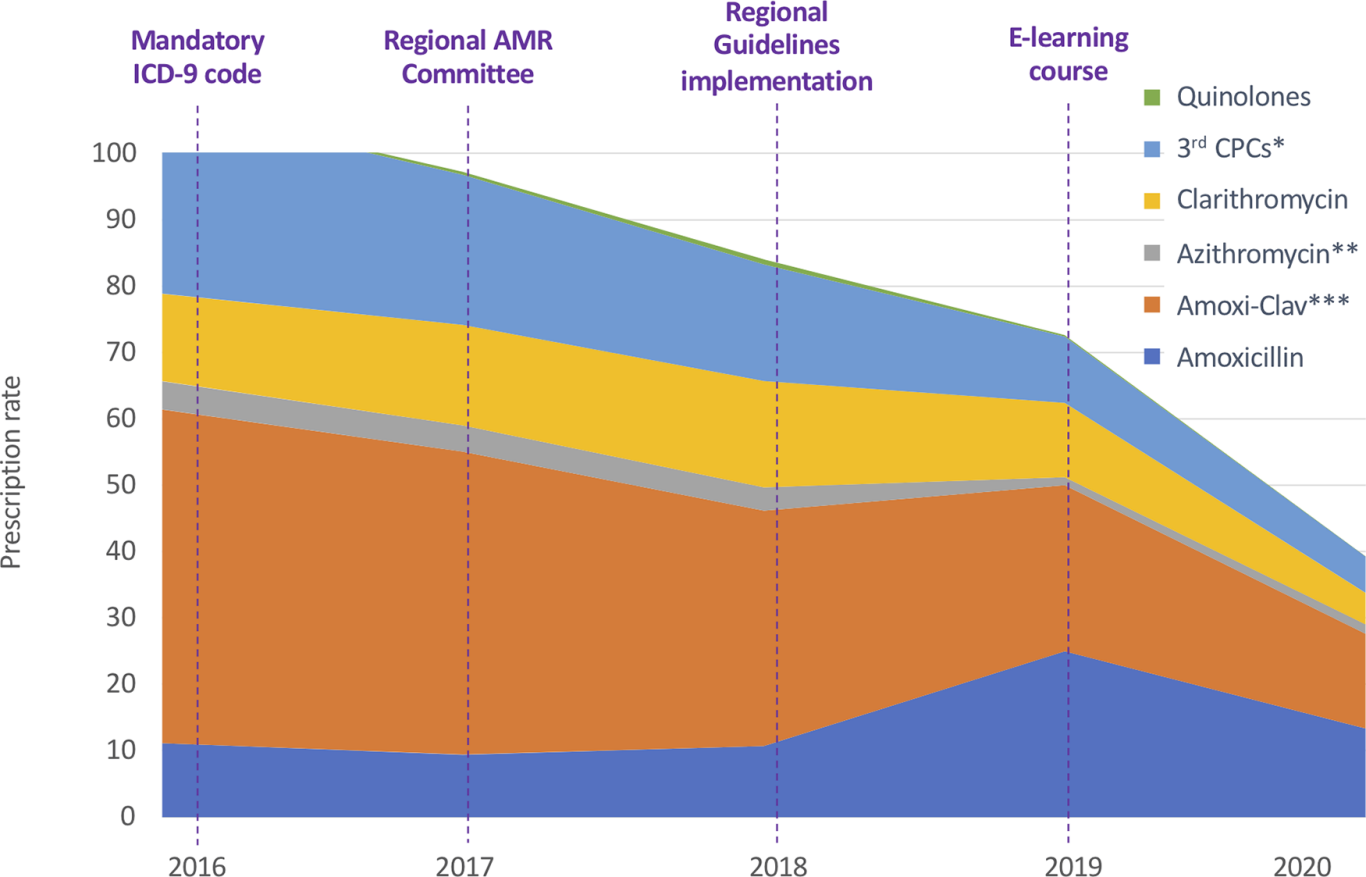
Annual antibiotic prescription rates for specific antibiotics classes and molecules. ICD-9: International Classification of Diseases-9. AMR, Antimicrobial Resistance. 3rd CPCs: third generation Cephalosporins. * *p* < 0.001. ** *P* = 0.029. *** *p* = 0.001.

During the study period the prescription rate of third generation Cephalosporins significantly decreased from 28.43 to 5.43 (*R*^2 ^= 0.995, *p* < 0.001). The prescription rate of Amoxicillin-Clavulanate also significantly decreased from 50.25 to 14.21 (*R*^2 ^= 0.983, *p* = 0.001) (Figure 2 and Supplementary Table S1).

Prescription rates of Amoxicillin (from 11.13 to 13.39; *R*^2 ^= 0.25, *p* = 0.391) and Quinolones (from 0.29 to 0.07; *R*^2 ^= .12, *p* = 0.572) did not change during the study period and we observed a trend toward progressive reduction in the prescription of Macrolides, although the difference in rates was not statistically significant (from 17.44 to 6.17; *R*^2 ^= 0.67, *p* = 0.091). As reported in Supplementary Table S1 the variations in the prescription rate reached statistical significance only for Azithromycin (*p* = 0.029).

**Figure 3:**
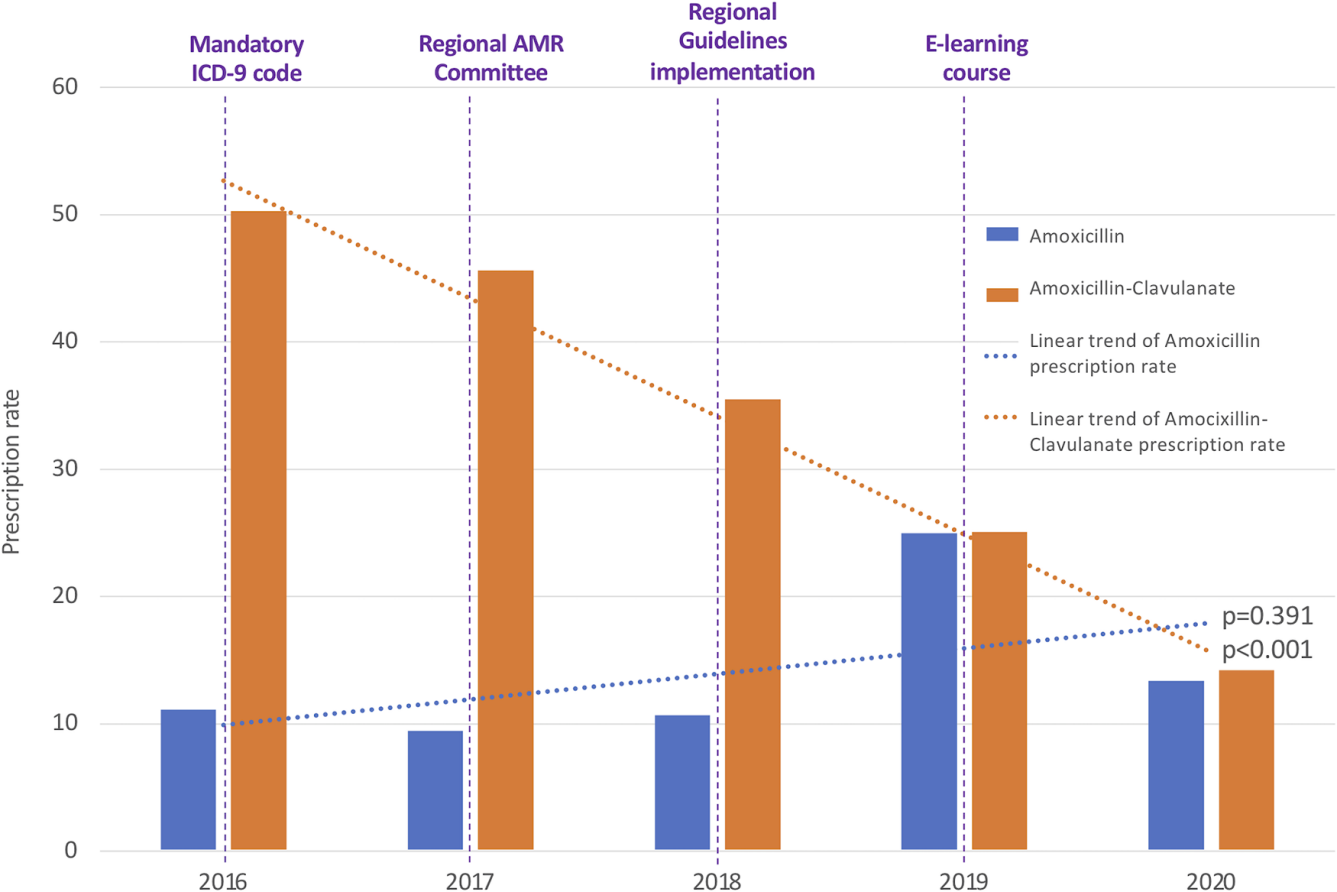
Annual antibiotic prescription rates of amoxicillin and amoxicillin-clavulanate. ICD-9, International Classification of Diseases-9; AMR, Antimicrobial Resistance.

A switch between the use of Amoxicillin-Clavulanate towards Amoxicillin occurred in parallel with the implementation of local guidelines and distribution of an e-learning course (Figure 3).

Regarding the analysis of relative antibiotic prescriptions, Amoxicillin-Clavulanate and third generation Cephalosporins were found significantly decreased, while non-significant variations were registered for the other broad-spectrum classes. The annual consumption of Amoxicillin-Clavulanate expressed as percentage of the total consumption of antibacterials for systemic use decreased from 44.90% to 34.95% (*p* = 0.033), with a parallel increase of Amoxicillin from 9.95% to 32.94% (*p* = 0.045). The consumption of third generation Cephalosporins expressed as a percentage of the total consumption of antibacterials for systemic use has been found to be reduced from 25.39% in 2016 to 13.36% in 2020 (*p* = 0.007). The consumption of fluoroquinolones and macrolides went respectively from 0.26% to 0.18% (*p* = 0.824) and from 37.17% to 25.07% (*p* = 0.429) of the total consumption.

**Figure 4:**
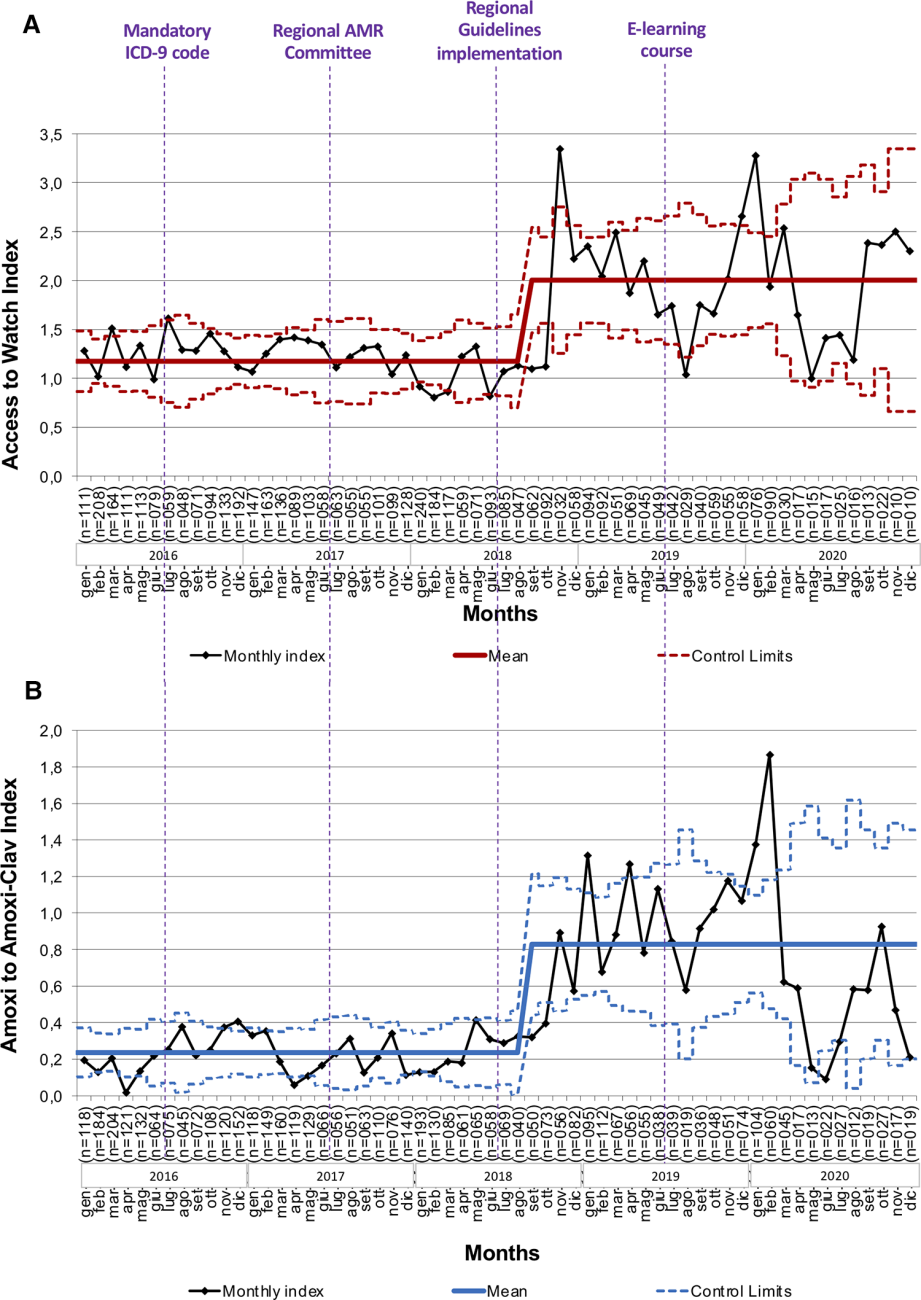
Trends of the access/watch and amoxicillin/amoxicillin-clavulanate indexes during the study period. (**A**) Access to Watch Index: monthly ratio between the prescription of Access and Watch antibiotics according to the *AWaRe* classification of the World Health Organization. (**B**) Amoxicillin to Amoxicillin-Clavulanate Index: monthly ratio between the prescriptions of Amoxicillin and Amoxicillin/Clavulanate. ICD-9, International Classification of Diseases-9; AMR, Antimicrobial Resistance.

Since September 2018, we have observed a significant change in the Amoxicillin/Amoxicillin-Clavulanate index from 0.24 (95%CI 0.006 to 0.467) to 0.83 (95%CI 0.510 to 1.149) (Figure 4 and Supplementary Figure S1). Similarly, the *Access/Watch* index increased from 1.17 (95%CI 0.700 to 1.648) to 2.00 (95%CI 1.464 to 2.542) after October 2018, following the spreading of the regional guidelines and the publication of an e-learning course (Figure 4 and Supplementary Figure S1).

We observed a seasonal variation in the antibiotic prescription rate per 100 patients, with a significant increase of overall prescriptions in Winter season (B = 4.405, 95%CI 3.292 to 5.717, *p* < 0.001).

### Impact of underlying conditions on the antibiotic prescription

The median number of antibiotic prescriptions was 2 (IQR 3–1) per patient annually. The presence of any underlying medical condition was not associated with a higher probability of antibiotic prescription. However, the presence of risk factors for recurrent UTIs (i.e., vesical-ureteral reflux or urinary tract malformation, *p* = 0.003) or the history of a previous episode of otitis media (*p* = 0.013), bronchitis (*p* < 0.001) and pneumonia (*p* = 0.026) were associated with a higher prescription frequency than the median value.

Patients who received more than 8 antibiotic prescriptions (3 IQRs above the median, *n* = 67) in at least one of the years of observation were identified as “outliers”. Among them we found a relatively high proportion of overweight or obesity (*n* = 16, 23.9%) that are the most frequent medical conditions beyond the history of previous infections.

In addition, we observed a significantly increased proportion of selected medical conditions in the group of outliers, including the presence of risk factors for recurrent UTIs (*p* = 0.039), asthma (*p* = 0.03) and autism (*p* = 0.005).

## Discussion

### Impact of the multifaceted intervention on the antibiotic prescription

Broad-spectrum molecules consumption in pediatrics is excessive, largely inappropriate and frequently associated with viral respiratory or intestinal illnesses (19, 20).

Following the implementation of a bundle of antimicrobial stewardship interventions, we observed a significant reduction in the overall antibiotic prescription rate per 100 patients in follow-up and per 100 medical consultations in a population of about 2,500 children followed for five consecutive years. In parallel, we recorded a massive reduction in the use of broad-spectrum molecules such as Amoxicillin-Clavulanate, third generation Cephalosporins and Azithromycin.

Inappropriate prescription in primary care is well documented by large-sample studies, such as the cross-sectional study by Butler et al. conducted in 14 primary care research networks in 13 European countries, that reported an antibiotic prescription in 50% of subjects seen for upper respiratory symptoms, with high variability in prescription frequency and molecules between different geographic areas (12). As an example, the proportion of broad-spectrum molecules in the metropolitan area of Milan (Italy) was substantially higher than the European average, with 38% of Macrolides and Lincosamides and 18% of Quinolones. The authors also observed a limited impact of the antibiotic prescription on the patients' clinical outcome, resembling a placebo effect. In our sample, the proportion of these antibiotics at the baseline were already very low, with Macrolides accounting for about 4% of total prescriptions and Quinolones less than 1%; yet, we observed a further decrease in prescription of these molecules.

The results of our study demonstrate that a multifaceted intervention addressed to PCPs is effective in improving local prescriptive habits. This evidence is in line with previous results obtained in Italian and European settings for other common pediatric diseases and treatments, supporting the hypothesis that targeting physicians working in primary care is an effective strategy to improve health-care and hence clinical outcomes. As an example, two studies conducted in outpatient settings showed how the implementation of educational face-to-face interventions (21) or e-learning courses (22) about the management of acute intestinal infections is a feasible and effective tool to enhance adherence to guidelines recommendations.

With specific regard to AMS programs, two randomized clinical trials published in JAMA (23) and Clinical Infectious Diseases (24), comparing antibiotic prescriptions between intervention and control group of PCPs, showed the effectiveness of an antimicrobial stewardship intervention on reducing antibiotic prescribing in pediatric outpatient setting and mostly the prescription of broad-spectrum antibiotics. Di Mario et al. assessed the effectiveness of a quality improvement project aimed at promoting a more rational antibiotic use in pediatric primary care in Central Italy, showing a significant reduction in the antibiotic consumption, a significant increase in the ratio of Amoxicillin to Amoxicillin-Clavulanate and a significant decline in the prescriptions of other second-choice antibiotics (25). The Sardinia region implemented an antimicrobial stewardship program comparable to what we reported in Campania. Although the results of this intervention have not been described, a contraction of the antibiotic consumption in Sardinia was documented in the 2019 National Report on antibiotics use (4, 26). The need for complex quality improvement programs is also confirmed by the outcomes of an EU-financed project (27, 28), which consisted of training courses on management of respiratory tract infections (RTIs), dissemination of clinical guidelines with recommendations for diagnosis and treatment, posters for the waiting rooms, brochures to patients and implementation of point of care tests to be used in the general surgeries. Reduced antibiotic prescriptions were reported immediately after the intervention with a slight increase in prescription rates six years after the implementation strategy, while antibiotics were more frequently prescribed by the general practitioners allocated to the control group.

The ESAC recommends the use of specific quality indicators for monitoring the antimicrobial drugs consumption, grouped into four clusters: consumption, relative consumption, broad/narrow, seasonal variation. The Defined Daily Dose (DDD)/1,000 patients' days is the most widely accepted method of consumption monitoring in adult specialties. However, this is inaccurate for monitoring antibiotic consumption in pediatric age range. Until today, there is no consensus on the best approximation method to use in pediatrics: the monitoring of days of therapy (DOT) per 100 or 1,000 patient days seems to be suitable, since it is not biased by the body weight variability (29–31).

For this reason and in line with most recent antibiotic reports in pediatrics (5, 23–25), we decided to express the first class of indicators as overall and specific prescription rates. Concerning the third class of indicators, we decided to describe the ratio between broad spectrum and narrow spectrum molecules using the Amoxicillin/Amoxicillin-Clavulanate index and Access/Watch index, in the purpose of comparing our research with other ones conducted in similar settings and in the same study period. Two Italian studies based on the Pedianet network recorded from PCPs between 2019 and 2021 showed a reduction in the overall prescriptions ranging from 20% to about 80% (32, 33). This data is consistent with the reduction observed in our study population, where the prescription rate dropped by about 65% of pre-implementation. In addition, they assessed the antibiotic prescription pattern analyzing the trend in slope of Amoxicillin/Amoxicillin-Clavulanate index and Access/Watch index, which did not show significant changes. We tested the effectiveness of the regional AMS interventions through the same parameters, and observed a more pronounced improvement of judicious use of antibiotics indexes after 2018, following the introduction of regional guidelines and empiric antibiotic therapy schemes and their dissemination through online educational interventions; indeed, a significant increase in *Access/Watch* antibiotics prescription index was registered since October 2018, according with a significant improvement of Amoxicillin/Amoxicillin-Clavulanate index since September 2018.

We complied with ESAC guidelines to assess the second and the fourth clusters of indicators, finding respectively significant decrease in the relative consumption of Amoxicillin-Clavulanate and third generation Cephalosporins and the presence of seasonal variation in overall antibiotic prescribing.

The reported change in the antibiotic prescription during the study period did not show significant harms in the study population. Although a retrospective analysis of the impact of the AMS interventions on the study population was not feasible, the pediatricians taking part in the study did not report a relevant difference in the access to Emergency Department or hospital admission during the five years of observations.

Finally, an additional key determinant of antibiotic prescription is the lack of communication between healthcare workers and patient/caregivers, that leads to repeated medical consultations and eventually results in an inappropriate prescription to meet expectations, as emphasized by the European Center for Diseases Prevention and Control (ECDC) in an informative pamphlet addressed to PCPs (34–38). Nevertheless, in comparison with the primary care setting in Europe, the presence of the PCP in Italy can be considered as a powerful tool in the organization of healthcare to achieve an optimal communication with families, offering to physicians a chance to break down wrong medical practices resulting from errors or misunderstanding with the families and reassure the latter about the limited risk of severe course of respiratory infections particularly in children.

### Impact of past medical history on the antibiotic prescription

Although we did not find a significant association between obesity and a high prescriptive frequency, within the outliers for number of prescriptions in a year obesity was among the most frequent conditions, together with previous episodes of respiratory tract infections (39–41).

While the relationship between the antibiotic prescription and the ongoing illness is well studied, there is lack of evidence about the relationship between the antibiotic prescription frequency and the past medical history and chronic conditions of the patient.

### Impact of COVID-19 pandemic on the antibiotic prescription in the primary care

In our cohort, we did not observe a significant variation in antibiotic prescriptions attributable to COVID-19 pandemic. From March to May 2020, during the first lockdown, there was a reduction of medical consultations, which were partially replaced by telephone consultations and other forms of telemedicine mainly for children with SARS-CoV-2 infections or other mild forms of respiratory illnesses. This, along with the first schemes proposed for COVID-19 treatment, could result in a possible increase in prescription of broad-spectrum molecules. To limit this effect, we standardized the prescription rates of our cohort either for 100 patients in follow-up or for 100 medical visits, but even with this approach we did not observe a change in the trend toward reduction of prescription started in 2018.

Although a large increase in the consumption of macrolides (especially Azithromycin) with limited evidence of efficacy (42, 43) has been described in the overall population during COVID-19 pandemic, we found a decreasing trend of Macrolides overall in our cohort. Azithromycin prescription showed a significant decrease during the study period, reaching the nadir in 2019 although showing a slight increase in the first semester of 2020, as a possible and transitory consequence of the misleading indications circulating during the first months of COVID-19 pandemic.

Some studies speculate that a similar effect could be explained by the rising use of remote medical consultations, registering variations in prescriptive frequency based on different socio-economic settings (44).

### Study limitations and future directions

Due to limitations in the data deductible from the practice management software, we analyzed the prescription rate instead of the days of therapy (DOT), which is more widely accepted as a method of quantification of antibiotic prescriptions in children (29–31). Nevertheless, we found that antibiotic prescription rates have already been used as an outcome of many renowned studies in literature (5, 23–25).

For the same reason we couldn't analyze factors related to the patient's clinical examination at the time of the prescription. As observed by O'Brien et al. (45) some clinical predictors of antibiotic prescription can be characterized, such as the presence of earache, breathing difficulty and poorer sleep and abnormal clinical examination of ear, throat or chest. In addition, all emotional and socio-psychological determinants of antibiotic prescriptions related to either the physicians and caregivers were inevitably not considered. Indeed, our cohort comes from a typical setting with low socio-economic status and educational level.

The data reported in the present study focused on the impact that the regional interventions had in a cohort of PCPs and do not apply to inpatient settings. A new version of the regional guidelines for empiric antibiotic prescription specifically addressed to hospital practitioners is going to be implemented at the end of the year. We believe that only a parallel implementation of AMS interventions, both in the outpatient and inpatient setting, could effectively impact on prescription habits and local AMR.

Finally, we believe that a longer follow-up period after implementation of AMS interventions could help to better understand not only their efficacy, but also their duration over time, and potentially to plan a catch-up intervention.

Antimicrobial resistance is an actual and challenging issue in pediatric healthcare.

Monitoring the consumption of antibiotics and the impact of antimicrobial stewardship policies is mandatory. In the light of a high geographical variability of AMR rates, local health policies and training programs should be promoted and tailored at local level. Additional steps for implementation should include: disseminating similar interventions to health-care practitioners working both in inpatient and outpatient settings, planning catch-up training programs to reinforce the adherence to national and local guidelines. Further research is required to understand the patients-related factors linked to a high frequency of prescriptions in order to better address these interventions.

## Data Availability

The raw data supporting the conclusions of this article will be made available by the authors if requested, without undue reservation.
