# Supplementary material for: Reduction in broad-spectrum antimicrobial prescriptions by primary care pediatricians following a multifaceted antimicrobial stewardship program
Source: Front Pediatr. 2023 Jan 6;10:1070325. doi: 10.3389/fped.2022.1070325 (PMC9853423; doi:10.3389/fped.2022.1070325)
Supplement: Supplementary file 1 [file Table1.docx]

Supplementary Material

This supplementary material is part of a study entitled “Reduction in broad-spectrum antimicrobial prescriptions by primary Care Pediatricians following a multifaceted Antimicrobial Stewardship program”. The study investigates the effect of local Public Health interventions, such as the publication of empiric antibiotic-therapy schemes and the implementation of an e-learning course, on the prescription rates of antibiotics and especially broad-spectrum classes and molecules, by three Primary Care Pediatricians of a single Sanitary District in the province of Naples, Italy.

**Supplementary Index:**

1. **Supplementary Tables**
2. **Supplementary Figures**

## Supplementary Tables

Supplementary Table 1. Linear regression of prescription rates of selected antibiotics

|  | 2016 | 2017 | 2018 | 2019 | 2020 | p |
| --- | --- | --- | --- | --- | --- | --- |
| Amoxicillin | 11,13 | 9,44 | 10,67 | 24,99 | 13,39 | 0,391 |
| Amoxicillin-Clavulanate | 50,26 | 45,62 | 35,51 | 25,06 | 14,21 | 0,001 |
| Cephalosporins, 3^rd^ gen | 28,43 | 22,64 | 17,5 | 9,95 | 5,43 | <0,001 |
| Azithromycin | 4,25 | 3,91 | 3,51 | 1,13 | 1,41 | 0,029 |
| Clarithromycin | 13,19 | 15,12 | 16,02 | 11,22 | 4,76 | 0,159 |
| Quinolones | 0,29 | 0,41 | 0,81 | 0,25 | 0,07 | 0,572 |

## Supplementary Figures


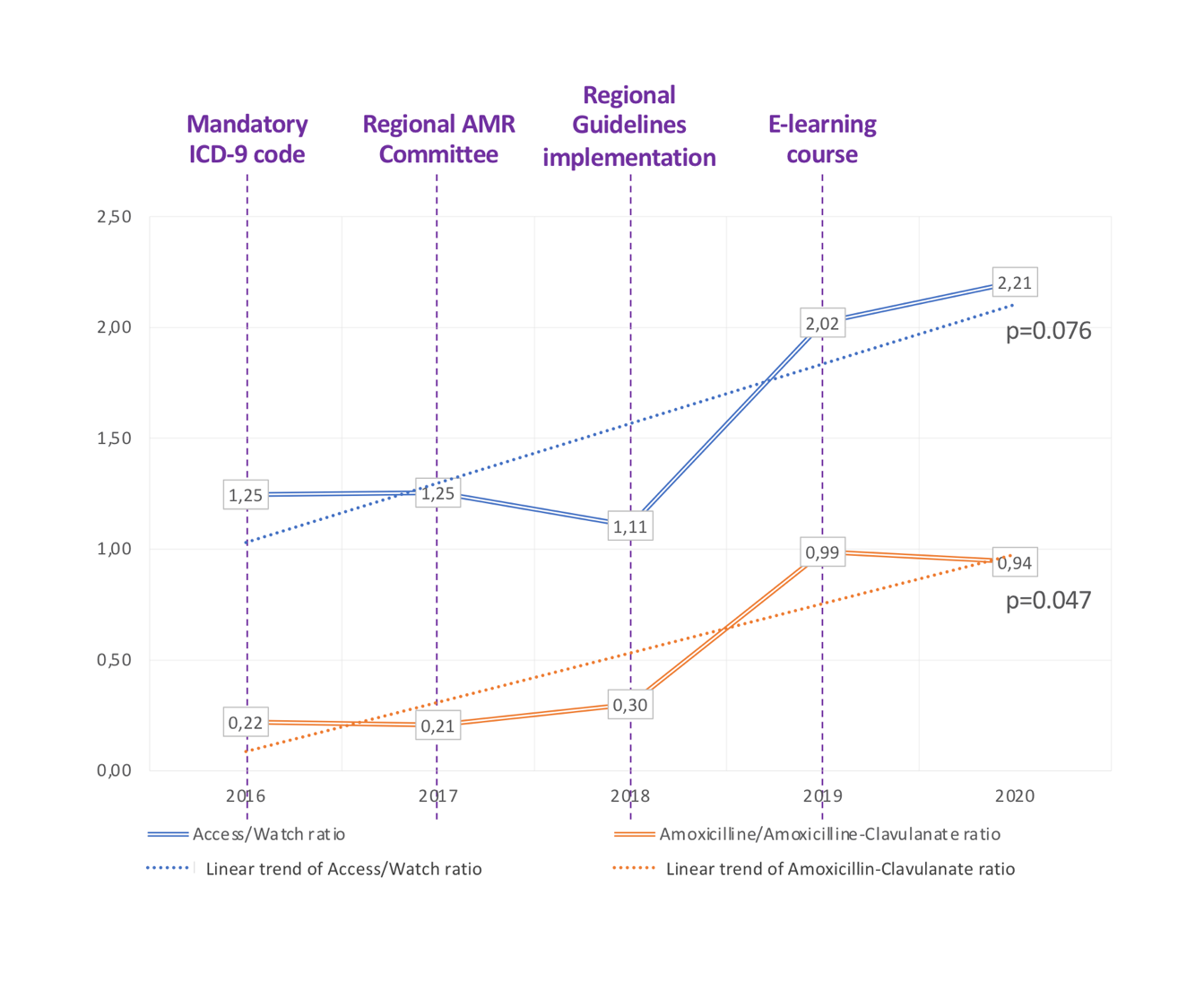


**Supplementary Figure 1. Trends of the *Access/Watch* and Amoxicillin/Amoxicillin-Clavulanate indexes during the study period**

ICD-9: International Classification of Diseases-9

AMR: Antimicrobial Resistance

Access/Watch ratio: annual ratio between the prescription of *Access* and *Watch* antibiotics according to the *AWaRe* classification of the WHO (11)

Amoxicillin/Amoxicillin-Clavulanate ratio: annual ratio between the prescription of Amoxicillin and Amoxicillin/Clavulanate
